# Preparing O/W/O Emulsion for Curcumin (*Curcuma longa*) Delivery and In Vitro Digestibility Assay

**DOI:** 10.3390/ijms26125639

**Published:** 2025-06-12

**Authors:** Kristýna Opustilová, Barbora Lapčíková, Daniela Sumczynski, Richard Adámek

**Affiliations:** Department of Food Technology, Faculty of Technology, Tomas Bata University in Zlin, Nam. T. G. Masaryka 5555, 760 01 Zlin, Czech Republic; kristyna.opustilova@gmail.com (K.O.); sumczynski@utb.cz (D.S.); richard.adamek@seznam.cz (R.A.)

**Keywords:** curcumin, emulsions, stability, particle size, digestibility, encapsulation, curcuma root powders

## Abstract

In this study, simple oil-in-water emulsions (O/W) and multiple O/W/O emulsions were employed as carriers for a curcumin delivery system. The stability of emulsions was evaluated using DSC (differential scanning calorimetry), accompanied by particle size measurement by DLS (dynamic light scattering) and rheological analysis. The amount of freezable water (W_fs_) in O/W emulsion was determined to be 80.4%, while that in O/W/O emulsion was 23.7%. Multiple emulsions had a more complex structure than simple emulsions, being characterized by higher stability with predominant loss modulus over storage modulus (G” > G’). The mean surface diameter for O/W emulsion was 198.7 ± 9.8 nm, being approximately two times lower than that for multiple emulsions. Curcumin in vitro digestibility was observed for both emulsions and, additionally, the digestibility of fresh and dried curcuma root powders was investigated. Multiple emulsions were found to be a superior matrix for curcumin delivery, with higher stability and emulsion digestibility of 50.6% for the stomach and small intestine. In vitro digestion of dried curcuma powders and curcuma root samples was monitored by HPLC (high-performance liquid chromatography). The DMD (dry matter digestibility) for dried curcuma powders ranged between 52.9% to 78.8%, and for fresh curcuma (KF) was 95.5%.

## 1. Introduction

Curcuma (*Curcuma longa*), commonly known as Indian saffron, is a member of the *Zingiberaceae* family, also known as the ginger family. This plant is primarily cultivated in the warm and humid climates of South Asian countries, such as China, Taiwan, India, Singapore, and Indonesia [1,2]. The health benefits of curcuma have been known for thousands of years, giving it a long history of use as a traditional medicine. Due to its health benefits, curcuma is used worldwide in various forms. For instance, in India, it is added to curry spice blends, in Japan, it is served in tea, in Thailand, it is used in cosmetics, in China, it serves as a dye, and in Malaysia, it is employed as an antiseptic [3]. The rhizome is the main source of phytochemicals called curcuminoids. Curcuminoids are polyphenolic compounds that give curcuma its characteristic colour and constitute about 3–6% of the rhizome’s weight, depending on the variety, soil, and climatic conditions. The most abundant curcuminoid in curcuma is curcumin, which makes up 70–77% of the total content. Other significant curcuminoids include demethoxycurcumin (15–20%) and bisdemethoxycurcumin (5–10%) [4]. As reported by Kim et al., cyclocurcumin is another curcuminoid contained in curcuma powder [5]. According to Regulation (EC) No 1333/2008 of the European Parliament and the Council, as amended, all curcuminoids are labelled with the collective name curcumin and coded E 100, and their use is not limited to food products. Curcumin plays a crucial role in reducing inflammation, preventing atherosclerosis, decreasing blood clot formation, and inhibiting the growth of *Helicobacter pylori* bacteria, which causes stomach ulcers. It can also bind with certain heavy metals, such as cadmium and lead, thereby reducing their toxicity [6]. The extensive list of curcumin health benefits has led to the development of new products in the food, pharmaceutical, and cosmetic industries [4].

Despite its promising potential in treating various diseases, the effectiveness of curcumin is limited due to its low bioavailability [7]. Curcumin is hydrophobic, resulting in extremely poor solubility in water. Its solubility is highly pH dependent, being practically insoluble at acidic and neutral pH levels. The ability to dissolve in gastrointestinal fluids is crucial for the absorption of any orally administered drug. Even with high oral doses of curcumin, its plasma concentration remains very low, as demonstrated in a clinical study. When administered doses as high as 12 g, the plasma concentration of curcumin was not sufficiently high to be detected [8,9,10]. Another significant factor is permeability, as drug absorption depends on its ability to penetrate the biological membrane of the gastrointestinal tract after dissolution [11]. Encapsulation of curcumin represents an effective strategy for enhancing its stability, solubility, and bioavailability, thereby potentially amplifying its therapeutic effects and application in the treatment of various diseases [12]. The most commonly used methods for curcumin encapsulation include (i) encapsulation in liposomes, (ii) encapsulation in micelles [13], which increases gastrointestinal absorption, and (iii) encapsulation using nanoemulsions or nanoparticles, which offer the main advantages of controlled particle size and properties, as well as gradual release [14].

This study focuses on the use of simple and multiple emulsions as carriers for curcumin and examines the differences in their digestibility. Another objective is to compare the digestibility and retention factors of fresh and dried curcuma roots.

## 2. Results and Discussion

### 2.1. Particle Size of Emulsions

**Figure 1:**
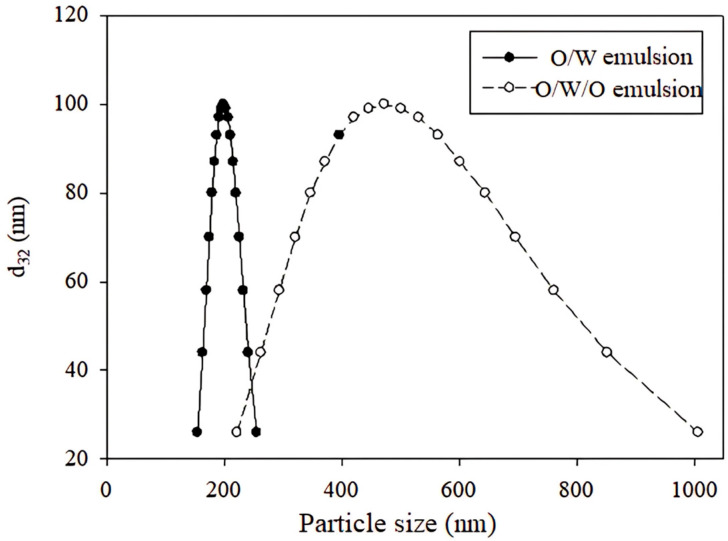
Distribution of particle sizes of the inner phase emulsion (O/W) (solid symbols) and multiple emulsion (O/W/O) (empty symbols).

The particle size distribution of the emulsions was measured using dynamic light scattering, and the results of surface-weighted mean diameter d_32_ are shown in Figure 1. As evident from the size distribution observed for O/W, it was a relatively monodisperse system, which is supported by the detected polydispersity index value of 0.043. This indicates that the particles in the system have very similar sizes; the mean surface diameter d_32_ measured for the inner phase was 198.7 ± 9.8 nm. The multiple emulsion (O/W/O) exhibits greater heterogeneity, with a polydispersity index value reaching 0.256. This indicates a wider range of particle sizes within the system. The mean surface diameter for the multiple emulsions was 472.8 ± 11.2 nm. The particle size in the multiple emulsions was approximately twice that of the simple emulsion, which can be attributed to the homogenization process and also to the limitations of the DLS technique applied. The O/W emulsion was homogenized at 25,000 RPM, whereas the multiple emulsions were homogenized at 10,000 RPM. Lower speeds were chosen to maintain the structure of the multiple emulsions. Higher speeds could potentially rupture the encapsulating membrane of the inner phase and destabilize the multiple emulsion system [15]. These findings feature the importance of the homogenization process in controlling the particle size distribution and stability of multiple emulsions, being crucial for their applications in various fields including food, pharmaceuticals, and cosmetics [16].

### 2.2. Viscoelatic Properties of Curcumin Emulsions

**Figure 2:**
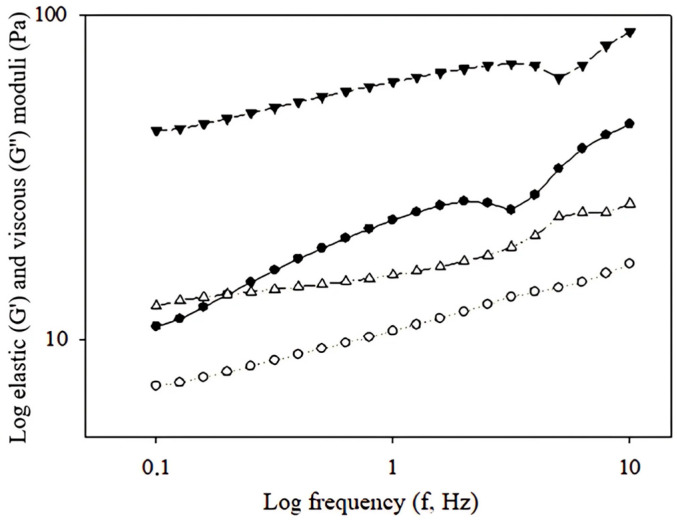
Viscoelastic properties of O/W emulsion (circles) and double O/W/O emulsion (triangles) (storage modulus G’, open white symbols, Pa; loss modulus G”, closed black symbols, Pa) as a function of frequency (f; ranging from 0.1 to 10.0 Hz).

Storage (G’) and loss (G”) moduli for simple emulsion and multiple emulsion as a function of frequency (0.1–10.0 Hz) are shown in Figure 2. Both tested samples exhibited predominantly loss modulus G” > G’, the O/W were significantly lower (*p* < 0.05) compared to those for the O/W/O. Multiple emulsions show a more complex structure than simple emulsions [17,18], which can lead to various interactions between the droplets. This may cause the viscous component to prevail over the elastic component. The loss modulus for multiple O/W/O emulsions showed the value of 74.14 ± 1.53 Pa detected at the frequency of 1 Hz. The value of the storage modulus for the O/W/O emulsion was almost three times lower, reaching 24.64 ± 0.66 Pa. The multiple emulsions showed a significant increase in the loss modulus, leading to a more fluid emulsion. It was observed that the higher the ratios of the oil phase in the external phase, the more fluid the emulsion became [19]. In this context, the droplets in the emulsion behaved more like a “liquid”, and their movement was governed more by viscous properties than by elastic ones, which led to the G” is greater than the G’. The rheological profile of O/W/O can be related to the content of κ-carrageenan, which led to the formation of high internal phase emulsion (HIPE), characterized by higher stability [20].

### 2.3. Differential Scanning Calorimetry

**Figure 3:**
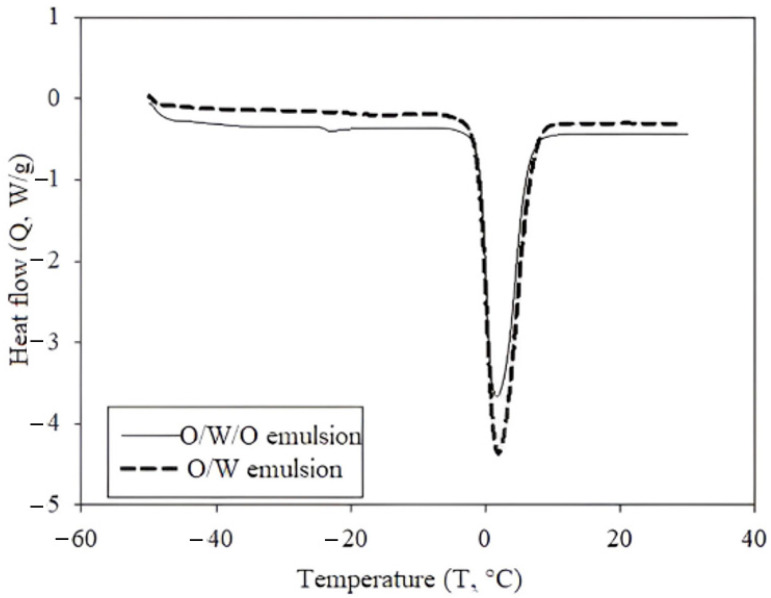
DSC curve of heating cycle with endothermic peak, which represents melting of freezable water in the multiple emulsion O/W/O (solid line) and in internal phase (simple emulsion) O/W (dashed line).

The DSC (differential scanning calorimetry) technique was employed to quantify the amount of water encapsulated in the form of droplets, and also to determine the quantity of the inner water phase in multiple emulsions [21]. The results for both simple (O/W) and multiple (O/W/O) emulsions are presented in Figure 3. The thermogram illustrates the melting enthalpy of freezable water, with the heating cycle following the cooling employed to evaluate the results [22]. As observed, the enthalpy for the simple emulsion was higher compared to that of the multiple emulsions. Specifically, the measured enthalpy of the O/W emulsion reached 268.13 J/g, while the enthalpy of the O/W/O emulsion was more than three times lower, 78.89 J/g. The enthalpy values can be related to the ratio and size distribution of water droplets in the emulsions, as the amount of water is directly proportional to the frozen water mass [21], with the onset melting temperature for both emulsions observed at −1 °C. The enthalpy values were subsequently used to determine the amount of freezable water (W_fs_), which reached the O/W emulsion of 80.4%, while the O/W/O emulsion was reduced to 23.7%. This difference can be attributed to the compositional profile of multiple emulsions, where the aqueous phase is surrounded by an oil layer, thereby resulting in a tighter binding of the water phase within the emulsion structure. In this context, it can be assumed that the encapsulation of the inner phase within the complex structure of O/W/O emulsion enhanced the stability of the system.

### 2.4. Results of Curcumin Release During Simulated Digestion in Emulsion Matrices

**Figure 4:**
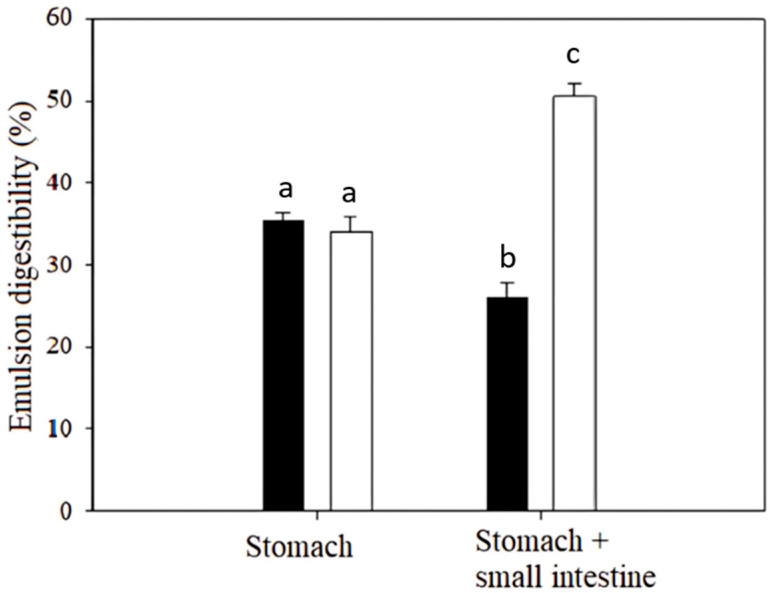
Digestibility of simple (black bars) and multiple emulsion (white bars) after the simulated stomach and stomach + small intestine digestion phases. Different superscript letters above the error bars indicate statistically significant differences (*p ≤* 0.05, Student’s *t*-test).

In this section, the ability of curcumin to release during simulated in vitro digestion was evaluated in both simple (O/W) and multiple (O/W/O) emulsions. The results of the analysis are presented in Figure 4.

The digestion simulation experiment showed that the release of curcumin varied depending on the type of emulsion. We observed that multiple emulsions for the stomach or both the stomach and small intestine showed more intense curcumin release than the simple emulsion type. The simple O/W emulsion released 35.3% of curcumin during the stomach digestion process, while the multiple O/W/O emulsion released 34.1% of curcumin from their original concentrations in the emulsions. During the stomach and small intestine digestion simulation, it was observed that the multiple emulsions released 50.6% of curcumin from its matrix, whereas the simple O/W emulsion released only 25.9%. These values were calculated based on the initial concentrations of curcumin in the emulsion samples.

The results suggest that the multiple O/W/O emulsion is more stable during digestion and can release a higher concentration of curcumin, which is then available for absorption processes in the gastrointestinal tract (GIT). In contrast, the simple O/W emulsion underwent degradation in the stomach, thereby reducing the efficiency of curcumin utilization by the human GIT. The degradation of the O/W emulsion and curcumin could be attributed to the low pH conditions in the stomach. The higher stability and digestibility of curcumin in the multiple O/W/O emulsion may be due to the encapsulation of curcumin inside the emulsion, which protects curcumin from rapid degradation in the stomach’s low pH environment. In the gastric phase, the multiple emulsion structure, particularly the outer oil phase and the interface stabilized by emulsifiers, provides initial protection to the inner phase, limiting premature curcumin release [23]. Upon reaching the small intestine, pancreatic lipases hydrolyze the lipids in the outer oil layer, and bile salts adsorb to the newly formed interfaces [24]. This enzymatic action and the presence of bile salts disrupt the O/W/O structure, leading to the breakdown of the intermediate water layer and subsequent release of the inner oil droplets and encapsulated curcumin into the intestinal lumen [25]. Additionally, partial degradation of multiple emulsions in the small intestine is desirable as it may facilitate curcumin release, thereby enhancing its availability for absorption through the intestinal epithelium It is known that curcumin stability is significantly influenced by pH; it is nearly insoluble in the acidic stomach environment but becomes more soluble with increasing pH (7.2–8.0), enabling absorption through the epithelium of the small intestine, whereas absorption does not occur in the stomach due to its insolubility. Therefore, losses of curcumin in the stomach are attributed to curcumin degradation [26]. This fact can be related to the prevalent release of curcumin from high-loaded nanoemulsions in the small intestine, as observed by Chen et al. [16]. From the results of our study, it can be inferred that curcumin encapsulated in the multiple emulsion may be more accessible for absorption in the GIT and appears to be a better option for protecting curcumin and increasing its availability for absorption compared to the simple emulsion.

The findings of our study can be compared with the data published for curcumin micro- and nano-formulations with enhanced GIT absorption [27]. The data of combined (stomach + small intestine) curcumin release are comparable with the intestinal release of curcumin from liposomal systems, determined in the range between 35 and 54% [28]. The values in our study are also in line with the percentage bioavailability of curcumin (observed after in vitro digestion) in Pickering emulsions stabilized by sunflower oil showing a bioavailability of 78.0%, and olive oil with 52.5% of curcumin bioavailability [29].

### 2.5. Effect of In Vitro Digestion on Curcumin Retention in the Matrix of Curcuma Root Samples

To illustrate the potential utilization of curcumin from natural sources during the digestion process, dried and fresh curcuma root samples were used. To determine retention factor values, it was necessary first to establish dry matter, ash content, and dry matter digestibility value of curcuma root samples, as presented in Table 1.

As can be seen in Table 1, the dry matter content ranged from 88.4 to 92.2% for powdered samples, while fresh curcuma root had a dry matter content of 16.9%. The ash content for dried samples reached 8.13%, while the fresh sample (KF) of curcuma had an ash content of 1.34%. Regarding Czech Reg. 398/2016, the curcuma powder can contain no more than 10% moisture and 9% of ash contents. It is evident from the results that samples K1 and K4 would not meet this moisture content value. The ash content was not exceeded in any powdered sample. Considering fresh curcuma sample KF, the dry matter and ash content significantly differ from powdered samples. The dry matter digestibility value was established using the enzymatic-gravimetric method employing *pepsin* and pancreatin enzymes. Considering the powdered types of curcuma samples, the DMD values ranged from 52.9 to 78.8%, and a fresh sample of curcuma root was 95.9% digestible. The highest DMD value of the powdered form of curcuma was observed for the domestic product K4 originating from Pakistan, whereas commercial curcuma powder samples exhibited lower digestibility, ranging between 52.9 and 59.6%. For comparison, Koláčková et al. [30] published digestibility values of various matcha teas ranging from 61.2 to 65.8%, while Sumczynski et al. [31] mentioned DMD values for uncooked wild rice samples in the range of 87.4 to 90.4%.

**Figure 5:**
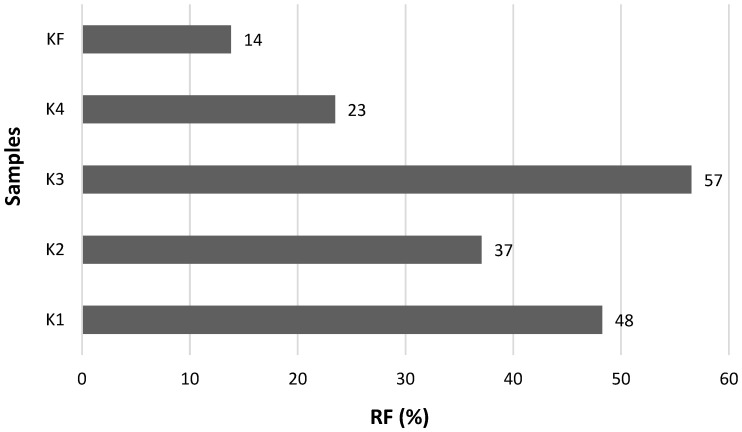
The remaining parts expressed as retention factor (RF, %) of curcumin complex after in vitro digestion of curcuma samples.

To determine the retention factor (RF), the data presented in Table 1 were computed by Equation (7), and the results are shown in Figure 5. It is a generally known fact that the matrix of food, technological processes, and the digestion process itself can modify the bioaccessibility and retention of substances from its matrix. It can be seen that the RF values for curcumin were determined in a wide range (from 14 to 57%). The lower the RF value, the lower the amount of curcumin remaining in the matrix of the undigested part of the curcuma roots, so it is potentially more accessible for absorption in the gastrointestinal tract [32]. The measured results showed that the most curcumin was released from the fresh root during digestion, when its retention was only 14%, which means that 86% of curcumin has been released, being bioaccessible for absorption by the human digestive tract. In the powdered form of curcuma root prepared at home (sample K4), curcumin was released from 77%, and its retention was 23%. Based on the results obtained, it can be hypothesized that after the industrial processing of curcuma root in powdered form, the retention of curcumin during digestion increases, and the curcumin is less released and therefore not as available for absorption by the GIT. Comparison of results with other studies is limited due to the scarce data provided under the same conditions for the digestion of curcuma root and the ability to obtain an undigested part of the roots for further analysis. This part of the experiment shows that curcumin is still a part of the undigested portion of the curcuma roots and, theoretically, could pass into the large intestine.

## 3. Materials and Methods

### 3.1. Material

Curcumin (95% total curcuminoids, Alfa Aesar, Karlsruhe, Germany), Nigella seed oil (100% BIO plant oil extracted from Nigella sativa, Nobilis Tilia s.r.o, Vlčí Hora, Czech Republic), κ-carrageenan (Sigma-Aldrich, Saint Louis, MO, USA); MW = 4.31 × 10^5^ Da, Czech Republic), phosphate buffer for particle size measurement (pH 7.0; Ing. Petr Švec—Penta s.r.o., Prague, Czech Republic), HCl (Sigma-Aldrich, Saint Louis, MO, USA), pepsin (Merck, JSC, Darmstadt, Germany, enzyme activity 0.7 FIP-U/mg), phosphate buffer for simulating small intestine conditions (pH 7.45; 3.09 g KH_2_PO_4_ and 32.49 g Na_2_HPO_4_ × 12H_2_O in 1.7 L distilled water), pancreatin (Merck, JSC, Darmstadt, Germany), enzyme activity: 350 FIP-U/g protease, 6000 FIP-U/g lipase, 75,000 FIP-U/g amylase), NaOH (Sigma Aldrich, Saint Louis, MO, USA), TWEEN 20 (M = 1.228 g/mol, (Sigma-Aldrich, Saint Louis, MO, USA), SPAN 80 (M = 460 g, (Carl Roth, Karlsruhe, Germany), hexane (Sigma-Aldrich, Saint Louis, MO, USA).

### 3.2. Preparation of Model Emulsion Samples

Two types of emulsions were prepared: simple oil-in-water (O/W) emulsion and multiple oil-in-water-in-oil (O/W/O) emulsion. The simple O/W emulsion (inner phase) was prepared as follows: by dissolving 1 g of curcumin in 10 mL of black cumin oil. The oil was stirred at 1000 RPM on a magnetic stirrer (IKA Rh basic2, Staufen, Germany) at a temperature of 50 ± 1 °C to ensure the complete dissolution of the curcumin for at least 1 h. Subsequently, the aqueous phase (W) was prepared by dissolving 0.9 mg of κ-carrageenan as a stabilizer in 90 mL of distilled water. The solution was thoroughly mixed and left to hydrate overnight at 4 ± 1 °C. To the κ-carrageenan solution, the 0.5 mL of emulsifier TWEEN 20 was added and the mixture was stirred on a magnetic stirrer for at least 1 h at 23 ± 1 °C. Then, the oil phase (O_1_) and the aqueous phase (W) were mixed in a ratio of 1:9 and subjected to homogenization using a homogenizer (SilentCrusher M, Heidolph Instruments GmbH and Co. KG, Schwabach, Germany) at 25,000 RPM for 5 min. This process resulted in a simple emulsion (O/W), which was used as the inner phase. The multiple emulsion (O_1_/W/O_2_) was formed by dispersing the inner emulsion phase (O/W) in the oil phase (O_2_). First, the emulsifier SPAN 80 (0.5 mL) was dissolved in 60 mL of coconut oil (Fair Trade BIO virgin coconut oil, Purity Vision, Koryčany, Czech Republic). The solution was stirred for at least 1 h at room temperature on a magnetic stirrer. Subsequently, 40 mL of O/W emulsion was added dropwise to the oil phase (O_2_). The emulsion was further subjected to homogenization at 10,000 RPM for 5 min using a homogenizer. The final emulsions were stored at 4 ± 1 °C for further analysis. The composition of the inner phase and O_1_/W/O_2_ per 100 mL is presented in Table 2.

### 3.3. Particle Size Measurement

Particle size was determined by measuring dynamic light scattering using the ZetaSizer (Brookhaven Instruments, New York, NY, USA) at a fixed angle of 90°, a refractive index of 1.3350, and a wavelength of 658 nm. To eliminate the effect of multiple scattering, the samples were diluted 100 × in phosphate buffer (pH 7.0). The intensity of the scattered light was converted to particle size using the Stokes-Einstein equation. The recorded values were expressed as the mean surface diameter (d_32_) from 5 repetitions. The particle size distribution in the inner phase of the emulsion (simple emulsion O/W) and the multiple emulsion (O/W/O) was analyzed as surface-weighted mean diameter d_32_ which is often used to evaluate the droplet size of freshly prepared emulsions because it can best reflect the emulsifying activity related to the number of interfaces generated [33].

### 3.4. Dynamic Oscillatory Rheology

An oscillatory shear rheometer, Kinexus (Malvern Panalytical Ltd., Malvern, UK), with a plate–plate geometry (parallel plate diameter of 40 mm and gap 1 mm), was used to analyze the viscoelastic properties of emulsions. The measurements were conducted at 20.0 ± 0.1 °C. To determine the linear viscoelastic region, a stress sweep was performed between 0.1 and 100 Pa at a frequency of 1 Hz. Measurements were carried out in oscillatory mode within the linear viscoelastic region with a shear stress amplitude of 1.0 Pa for simple emulsion and 20.0 Pa for multiple emulsion, and for both emulsion types in the frequency range from 0.1 to 10.0 Hz. Storage (G’, Pa) and loss (G”, Pa) moduli were recorded as functions of frequency f (Hz) using the rSpace software (Malvern Panalytical, version 1.17.2398).

### 3.5. Determination of Freezable Water Content in Emulsions by DSC

The amount of freezable water emulsions was monitored using the differential scanning calorimeter DSC 250 (TA Instruments, New Castle, DE, USA), following the procedure by Dalmazzone et al. [22]. The emulsion 15.0 ± 0.5 mg was weighed into a hermetically sealed pan. The experimental conditions were set as follows: a cooling process from +25 to −50 °C, at a cooling rate of 10 °C/min, maintaining an isothermal process for 1 min, followed by a heating process from −50 °C to +30 °C, at a heating rate of 5 °C/min. All experiments were conducted in an inert nitrogen atmosphere (gas flow rate of 50 mL/min). An empty pan was used as the reference. To interpret the measured data, the baseline was subtracted from the measured signal. The energy required for the phase transition was determined by integrating the area under the corresponding peak in the thermogram using the TRIOS 5.7.0.56 software (TA Instruments, New Castle, DE, USA). The amount of freezable water (W_fs_, %) was calculated according to Equation (1):

(1) $$W_{fs}\left(\%\right)=\frac{\Delta H_{e}}{\Delta H_{ice}}\times 100$$

where ΔH_e_ is emulsion enthalpy (J/g) and ΔH_ice_ is the enthalpy of pure water 333.5 (J/g), as reported by Tylewicz et al. [34]. All experiments were performed in triplicate.

### 3.6. Release of Curcumin Complex Encapsulated in Emulsions During Simulated Digestion In Vitro

The digestibility of curcumin encapsulated in simple (O/W) and multiple (O/W/O) emulsions under in vitro conditions was studied using a two-stage digestion process according to Zhou et al. [35] with minor modifications based on our knowledge. The first step of simulated stomach digestion was as follows: the emulsion (0.5 g) was weighed in a 50 mL Erlenmeyer flask, to which 20 mL of 0.1M HCl and 0.25 g of pepsin were added. The flasks were sealed and incubated in a shaking water bath (Memmert GmbH, Buchenbach, Germany) at 37 °C for 2 h. After simulating the digestion process in the stomach, both gastric juices and partially digested samples were transferred to a 100 mL Erlenmeyer flask to which 40 mL of small intestine simulation fluid composed of phosphate buffer (pH 7.45) and 0.25 g of pancreatic enzymes were added. Consequently, the flasks were sealed and incubated in a shaking water bath at 37 °C for 24 h. After the incubation period simulated digestion in the small intestine, the samples were mixed with 10 mL of hexane and shaken in a separator funnel to extract the curcumin. The hexane was then evaporated to dryness in ceramic dishes above the water bath at a temperature of 60 °C (GFL, Gujarat, India) and reconstituted with 10 mL of methanol [35]. The digestibility value of curcumin in the emulsions was measured in the native form of simple (O/W) and multiple (O/W/O) emulsions. The concentration of curcumin released (digested) during simulated in vitro digestion in the stomach and small intestine was determined using HPLC. All experiments were performed in triplicate.

### 3.7. Determination of Curcumin Content Using HPLC Method

The curcumin profile was analyzed using an HPLC Dionex Ultimate 3000 liquid chromatogram system equipped with the diode array detector DAD-3000 RS (Thermo Fisher Scientific, Waltham, MA, USA). The extracted complex of curcuminoids (demethoxycurcumin, bisdemethoxycurcumin, curcumin, and cyclocurcumin), expressed as the sum of curcumin, was filtered through a syringe nylon filter (13 mm, 0.22 µm) (Labstore, London, UK). The 5 µL volume of methanol extract was injected into a reverse phase Agilent ZORBAX Eclipse Plus C18 column (50 × 3 mm; 1.8 μm) (Agilent Technologies, Santa Clara, CA, USA). The elution was performed isocratically with a mobile phase consisting of acetonitrile and 2% acetic acid in a 40:60 ratio. The flow rate of the mobile phase was 0.5 mL/min, the column temperature was set at 33 °C, and the chromatogram was recorded at a wavelength of 420 nm. In the calibration range of 0–50.0 μg/mL, the DAD response was linear for all curcuminoids whereas correlation coefficients exceeded 0.9989. The individual compounds were identified according to the retention time obtained from the chromatogram and the method of standard addition. The content of each analyte was then calculated from the linear regression equation and expressed as the sum of curcumin in mg per 1 g of sample (mg/g) [36].

### 3.8. The Effect of Sample Matrix on Curcumin Release During Simulated In Vitro Digestion

#### 3.8.1. Preparation of Samples from Native and Undigested Parts of Curcuma Root

To determine the dry matter digestibility (DMD, %) and the retention factor (RF, %) values for the curcumin complex, four samples of dried and fresh curcuma root were used. The samples were labelled K1 (dried Bio curcuma spice, supported by Bio Nebio, Bavoryně, Czech Republic) and K2 (dried Bio curcuma spice, supported by Sonnentor, Čejkovice, Czech Republic) both originating from India. The K3 dried curcuma sample was purchased directly in India (Mida and Company Private Limited, Kolkata, West Bengal), and a homemade curcuma sample was designed as sample K4 (originating from Pakistan). A sample of fresh turmeric root labelled KF was purchased from a commercial store (Titbit, Praha, Czech Republic) and its country of origin was Thailand. To obtain solid undigested parts of the curcuma samples mentioned above, the digestibility assessment was completed after drying the samples at 35 °C for 24 h. To measure curcumin concentrations using HPLC, extracts from dried, fresh and undigested portions of curcuma samples were prepared as follows: an aliquot weight of 2.5 mg sample was weighed in a 50 mL volumetric flask that was made up to the mark with methanol. Subsequently, the flask was placed in a thermostatic ultrasound bath K2L (Kraintek Czech, s.r.o., Hradec Králové, Czech Republic) for at least 30 min at 25 °C to extract the curcumin complex. Finally, the methanolic extract was filtered through a nylon syringe filter (13 mm, 0.45 µm), and thus prepared for HPLC analysis [17].

#### 3.8.2. Determination of In Vitro Digestibility of Curcuma Samples

The in vitro digestion process was carried out in two stages, including digestion in the stomach with *pepsin* and then in the small intestine with pancreatic enzymes [32] using a Daisy^II^ incubator (Ankom Technology, Macedon, NY, USA). First, samples were weighed (0.25 g) and sealed in F57 Ankom Technology bags (Macedon, NY, USA) using an impulse sealer KF-200H (Penta Servis, Holice, Czech Republic). To simulate stomach digestion, the incubation bottle was filled with 1.7 L of 0.1 M HCl containing pepsin (1.88 g) with an activity of 0.7 FIG-U/mg. The samples were incubated for 2 h at 37 °C. Consequently, stomach solutions were drained, and samples sealed in bags were rinsed in redistilled water so that they could subsequently undergo digestion in the small intestine. The rinsing is not a direct physiological process, but it was applied to mimic the neutralization of gastric contents and the enzyme transition that occurs naturally [37]. This step helped to create a controlled environment for the subsequent intestinal digestion phase, ensuring that the conditions were optimal for the activity of pancreatic enzymes [38].

To simulate small intestine digestion, 1.7 L of phosphate buffer (pH 7.45) and pancreatic enzyme mixture (1.88 g), with an activity of 350 FIG-U/g protease, 7500 FIG-U/g amylase and 6000 FIG-U/g lipase, were added to the incubation bottle. After 24 h of incubation at 37 °C, the samples were rinsed multiple times with redistilled water, and the bags with the undigested sample fractions were dried at 105 °C for 24 h and weighed. Finally, the samples were combusted in a muffle furnace (LM112.10, Veb Elektro, Berlin, Germany) at 550 °C for 5.5 h, cooled, and weighed. The digestibility of the samples, expressed as the DMD value, was calculated using Equations (2)–(6):

(2) $$DMD\;\left(\%\right)\;=\;100\;-\frac{100\times \;DMR}{m_{2}\times DM}$$

(3) $$DMR\;\left(g\right)\;=\;m_{3\;}-\;m_{1}\times c_{1}$$

(4) $$DM\;\left(g\right)\;=\frac{\;DW\times m_{s}}{m_{1}}$$

(5) $$c_{1}\left(g\right)\;=\frac{\;m_{s}}{m_{1}}$$

(6) $$c_{2}\left(g\right)=\frac{m_{p}}{m_{1}}$$

where DMD is the dry matter digestibility (%), DMR is the mass of the sample without the bag after digestion and drying (g), DM is the mass of the dry matter of the sample (g), DW is the dry weight of the sample (expressed as %), m_s_ is the mass of the sample for dry matter determination (g), c_1_ is the correction weight of the bag after drying (g), c_2_ is the correction weight of the bag after combustion (g), m_p_ is the mass of ash of the empty correction bag (g), m_1_ is the weight of the empty bag (g), m_2_ is the sample weight (g), m_3_ is the weight of the dried bag with the sample after incubation (g).

#### 3.8.3. Evaluation of Retention Factor (RF) for Curcumin

To monitor the release of curcumin from the matrix of natural curcuma samples used as spices and fresh curcuma root, an in vitro digestion simulation technique was used followed by the quantification of curcumin in native and undigested forms of the samples using HPLC. The effect of the sample matrix on curcumin release during digestion is expressed by the retention factor (RF, %), which indicates the proportion of curcumin still bound in the matrix of the undigested fraction of the sample after simulating the digestion process in the stomach and small intestine. The retention factor can be calculated according to Equation (7):

(7) $$RF\left(\%\right)\;=\frac{concentration\;of\;curcuminin\;undigested\;sample\times \left(100\;-\;DMD\right)}{concentration\;of\;curcumin\;in\;native\;sample}$$

where DMD is the dry matter digestibility (%), as defined by Equation (2).

#### 3.8.4. Determination of the Dry Matter and Ash Content

The ash and dry matter contents were determined as supportive parameters for the subsequent calculation of the digestibility value. The determination of dry matter was performed using the reference gravimetric method EN ISO 712 (461014) [39] and the ash content was also determined gravimetrically according to the norm ISO 2171 [40].

### 3.9. Statistical Analysis

To assess the potential normality of the data, the Shapiro–Wilk test was performed with a significance level α set at 0.05. Subsequently, the results were statistically evaluated using analysis of variability (ANOVA) and Student’s *t*-test at a significance level of α = 0.05 [41]. Data processing was performed using SigmaPlot software version 12.5 (Systat Software, San Jose, CA, USA).

## 4. Conclusions

In conclusion, the findings from this study underscore the significant impact of emulsion type and preparation process on the particle size, stability, and curcumin release during simulated digestion. The comparison between the simple O/W emulsion and the multiple O/W/O emulsion revealed that the latter exhibited a broader particle size distribution, as reflected by a higher polydispersity index and a larger mean surface diameter. This increase in particle size in the multiple emulsions was likely attributed to the lower homogenization speed used, which preserved the structural integrity of the system. Furthermore, the rheological properties demonstrated that the O/W/O emulsion exhibited higher viscoelastic moduli, compared to simple emulsion, when the multiple emulsions exhibited a more fluid consistency. These differences in viscoelasticity behaviour corresponded to a more complex structure of multiple emulsions, which is important for functional applications of the emulsions in various industries, including food and pharmaceuticals.

Moreover, the analysis of curcumin release during simulated digestion provides valuable insights into the potential benefits of using multiple emulsions for enhancing curcumin bioavailability. The digestibility of curcumin in simple and multiple emulsions was significantly different. The multiple O/W/O emulsion showed a more stable release of curcumin, with a higher percentage of curcumin being available for absorption in the small intestine compared to the simple O/W emulsion. As observed during the stomach and small intestine digestion simulation, the multiple emulsions released 50.6% of curcumin, while the simple emulsion was two times less amount (25.9%). This finding is particularly beneficial for enhancing the bioaccessibility of curcumin, which is known to be poorly soluble in acidic stomach conditions. The protective effect of the multiple emulsions, which shields curcumin from degradation in the stomach, likely facilitates its release and absorption in the small intestine. In comparison, curcumin from the O/W emulsion was more susceptible to degradation in the acidic stomach environment, limiting its bioavailability. These findings suggest that the use of multiple emulsions can be a promising strategy for improving the therapeutic potential of lipophilic bioactive compounds by increasing their stability and enhancing their absorption in the gastrointestinal tract. While the in vitro digestion model provides valuable comparative insights into curcumin release from different formulations, future in vivo studies are substantiated to assess the curcumin bioavailability and physiological impact on the human body, including dynamic nutrient absorption.

## Figures and Tables

**Table 1:** Results of dry matter and ash content, digestibility, and curcumin concentrations.

Sample	Dry Matter (%)	Ash Content (%)	DMD ^1^ (%)	Curcumin in Native Part (mg/g)	Curcumin in Undigested Part (mg/g)
K1	88.4 ± 0.4 ^a^	8.13 ± 0.21 ^a^	56.2 ± 0.6 ^a^	33.4 ± 0.1 ^a^	36.9 ± 0.6 ^a^
K2	92.2 ± 0.2 ^b^	6.38 ± 0.01 ^b^	52.9 ± 1.1 ^b^	38.4 ± 0.1 ^b^	30.2 ± 0.1 ^b^
K3	90.0 ± 0.2 ^c^	5.05 ± 0.05 ^c^	59.6 ± 2.0 ^c^	24.1 ± 0.1 ^c^	33.6 ± 0.6 ^c^
K4	89.4 ± 0.2 ^d^	7.84 ± 0.03 ^d^	78.8 ± 2.1 ^d^	32.8 ± 0.1 ^d^	36.2 ± 0.1 ^a^
KF	16.9 ± 1.2 ^e^	1.34 ± 0.11 ^e^	95.9 ± 3.2 ^e^	6.95 ± 0.01 ^e^	23.3 ± 0.2 ^d^

The results are presented as mean ± SD (*n* = 3). Means within a column with identical superscript letters do not differ significantly (*p* > 0.05, Student’s *t*-test). ^1^ DMD—dry matter digestibility value.

**Table 2:** Composition of inner phase and multiple emulsion (O_1_/W/O_2_) /100 mL of sample.

Ingredients ^1^	O/W	Ingredients ^1^	O_1_/W/O_2_
Curcumin (g)	1.0	Inner phase (mL)	40
Black cumin oil (mL)	10.0	SPAN 80 (mL)	0.5
Distilled water (mL)	90.0	Coconut oil (mL)	60.0
TWEEN 20 (g)	0.5		
κ-carrageenan (g)	1.0		
**Processing Parameters**	**O/W**	**Processing Parameters**	**O_1_/W/O_2_**
Homogenization (RPM) ^2^	25,000	Homogenization (RPM) ^2^	10,000
Time of homogenization (min)	5.0	Time of homogenization (min)	5.0
Temperature (°C)	25.0	Temperature (°C)	25.0

^1^ Ingredient amount per 100 mL of emulsion. ^2^ RPM—rotations per minute.

## Data Availability

The raw data supporting the conclusions of this article will be made available by the authors upon request.

## Abbreviations

The following abbreviations are used in this manuscript:

DLS	Dynamic light scattering
DMD	Dry matter digestibility
DMR	Mass of the sample after digestion and drying
DSC	Differential scanning calorimetry
DW	Dry weight of the sample
FIG-U	Federation Internationale Pharmaceutique Units
GIT	Gastrointestinal tract
HIPE	High internal phase emulsion
HPLC	High-performance liquid chromatography
O/W	Oil-in-water emulsion
O/W/O	Oil-in-water-in-oil emulsion
RF	Retention factor
RPM	Rotations per minute
